# Lipase-Secreting *Bacillus* Species in an Oil-Contaminated Habitat: Promising Strains to Alleviate Oil Pollution

**DOI:** 10.1155/2015/820575

**Published:** 2015-06-09

**Authors:** Li Pin Lee, Hudzaifah Mohamed Karbul, Marimuthu Citartan, Subash C. B. Gopinath, Thangavel Lakshmipriya, Thean-Hock Tang

**Affiliations:** ^1^Advanced Medical & Dental Institute (AMDI), Universiti Sains Malaysia, 13200 Kepala Batas, Penang, Malaysia; ^2^Institute of Nano Electronic Engineering (INEE) and School of Bioprocess Engineering, Universiti Malaysia Perlis, 01000 Kangar, Perlis, Malaysia

## Abstract

Lipases are of great interest for different industrial applications due to their diversity and versatility. Among different lipases, microbial lipases are preferable due to their broad substrate specificity, and higher stability with lower production costs compared to the lipases from plants and animals. In the past, a vast number of bacterial species have been reported as potential lipases producers. In this study, the lipases-producing bacterial species were isolated from an oil spillage area in the conventional night market. Isolated species were identified as *Bacillus* species by biochemical tests which indicate their predominant establishment, and further screened on the agar solid surfaces using lipid and gelatin as the substrates. Out of the ten strains tested, four potential strains were subjected to comparison analysis of the lipolytic versus proteolytic activities. Strain 10 exhibited the highest lipolytic and proteolytic activity. In all the strains, the proteolytic activity is higher than the lipolytic activity except for strain 8, suggesting the possibility for substrate-based extracellular gene induction. The simultaneous secretion of both the lipase and protease is a mean of survival. The isolated bacterial species which harbour both lipase and protease enzymes could render potential industrial-based applications and solve environmental issues.

## 1. Introduction

Oil pollution or spillage is a prevalent problem in developing and industrialized countries. One of the major sources of oil pollution is the dietary oil spillage from both the producers and end-users, which is a very widespread form of pollution in the environment leading to a number of other complications such as the clogging of pipes and drainages. In order to alleviate the problem associated with this, degradation of the oil can be mediated with an environment-friendly technology and cost-saving system [1]. However, this cleaning-up system is dependent on several factors such as the nature of the place of oil contamination occurrence, temperature, as well as the microbial consortium. There are different microbial species reported from the oil-polluted environments, which include bacteria, fungi, and actinomyces [2–7]. Owing to the presence of a large diversity of microorganisms, one amenable approach to assuage this oil contamination-related problem is the microbial-based degradation. To expedite microbial-based degradation, the type of bacteria and the corresponding products secreted must be identified prior to the characterization. In addition to this, identification and characterizations are able to unveil the presence of a potpourri of extracellular enzymes, which can be purified. Previous studies revealed the presence of lipolytic bacteria isolated from different habitats such as industrial wastes, vegetable oil processing factories, dairy plants, and soil contaminated with oil, where these oily environments provide good conditions for these bacteria to flourish [8, 9]. For example, a novel species of lipase producing bacteria,* Geobacillus zalihae*, was isolated from the palm oil mill effluent in Malaysia [10].

**Figure 1:**
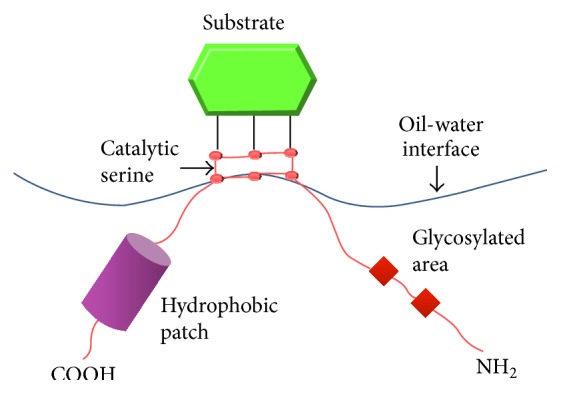
Hydrolysis of the triglyceride by lipase takes place at the interface of the insoluble substrate phase and the aqueous phase, the region where enzyme is dissolved.

**Figure 2:**
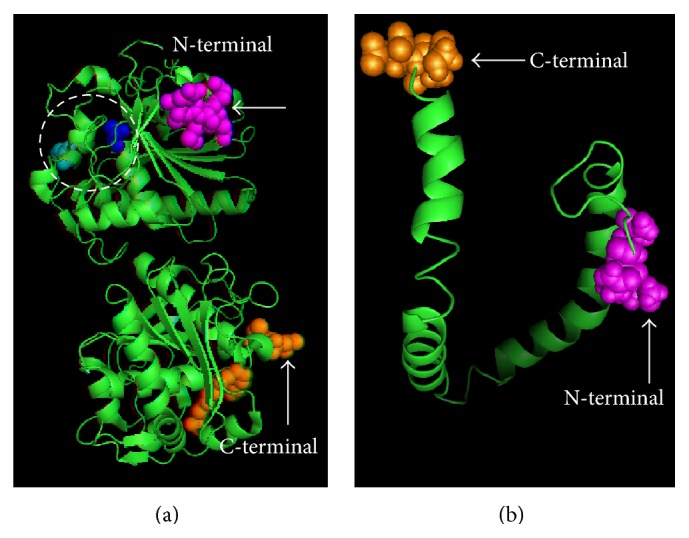
(a) Crystalized structure of* Thermomyces lanuginosa* lipase mutants TLL (S146A) and the substrate oleic acid (protein data bank accession number-1GT6). (b) Crystalized structure of apolipoprotein-SDS micelle complex (protein data bank accession number-1O8T).

One of the most common extracellular secretions by the bacteria-inhabiting oil-rich soil is the lipase. Lipases or triacylglycerol acyl hydrolases (E.C.3.1.1.3) are enzymes that catalyze the hydrolysis of ester bonds in fats and oils into glycerol and free fatty acids at the oil-water interface [9]. This hydrolysis takes place at the interface between the insoluble substrate phase and the aqueous phase, the region where the enzyme dissolves (Figure 1). Apart from hydrolysis, lipases also catalyze synthesis reactions, for example, esterification, amidation, alcoholysis, acidolysis, and aminolysis [11]. Yapoudjian et al. [12] have shown that the two possible binding modes of* Thermomyces lanuginosa* lipase mutants TLL (S146A) and the substrate oleic acid involve interaction with the tryptophan 89 of the lipase (protein data bank accession number-1GT6) (Figure 2(a)). Meanwhile, Zdunek et al. [13] have determined the global structure of apolipoprotein, the activator of lipoprotein lipase that forms complex with the sodium dodecyl sulfate (SDS) micelles (protein data bank accession number-1O8T) (Figure 2(b)).

The ability of lipases to catalyze reactions in a broad range of substrates without the addition of expensive cofactors and their stability in organic solvents resulted in the enzymes being listed as the third largest group of commercialized enzymes after proteases and carbohydrases [11, 14]. Lipases are commonly used in the food industry, pulp and paper processes, medical field, and as cleaning agents [14–16]. Lipases were produced naturally in several species of animals, plants, bacteria, yeasts, and fungi [17]. However, lipases isolated from bacteria have gained vast attraction due to their higher activities under optimum pH at neutral or alkaline condition. Microbial lipases also have shorter generation times, and genetic manipulations can be performed more easily on bacterial cells to increase the enzyme production [14]. Besides, bacterial cultures were more readily scaled up for production and purification with lower production costs [17, 18]. Due to the wide ranging versatility of lipases in biotechnological applications, the demands for new lipase sources continues to stimulate the screening and identification of novel lipolytic bacteria with the highest ability for the biodegradation of oils and fats [1, 19, 20]. Thus, in this study, samples from night market were chosen for screening lipase producing bacteria in the oil-contaminated soil, which can be potential microbial-based degradation approach and an “extraction pool” for the extracellular enzymes. Moreover, the differential secretion of the lipase and protease enzyme was also analyzed. Lipase and protease are the class of enzymes that perform both the degradative and synthetic function for the physiological necessity of the microorganism. The comparison can provide knowledge on the differential secretion of lipase/protease in the oil-contaminated soil. Moreover, the identification of the strains that excrete these enzymes can be also potentially manipulated by culturing them in media to expedite large scale enzyme production. These enzymes can also be useful to decontaminate the oil contaminants in the collection area of the night market that are very difficult to be cleaned.

## 2. Materials and Methods

### 2.1. Isolation of Bacteria from Oil-Contaminated Soil

Oil-contaminated soil samples were collected from the night market at Bandar Putra Bertam, Penang, Malaysia, and were processed immediately on the same day of collection. The soil samples (5%) were inoculated into enrichment medium (EM1) containing 1% olive oil and incubated at 60°C for 2 days under shaking condition as described by Abd Rahman et al. [10]. Enriched cultures were then streaked onto LB agar plate (Lennox) (Laboratorios CONDA, Spain) for isolation of single colonies.

### 2.2. Identification of Lipase Producing Bacteria

The bacterial isolates were grown in 10 mL of Luria Broth (Laboratorios CONDA, Spain) at 37°C overnight at 200 rpm and later subjected to Gram staining by using BD Gram Stain Kit (Becton, Dickinson and Company, USA). In order to identify the bacteria, the cultures were subjected to catalase, coagulase, oxidase, motility, indole, citrase, urease, methyl red, and Triple Sugar Iron (T.S.I) test. Prior to the lipolytic activity assay, the enriched cultures of ten bacterial strains were streaked onto blood agar plates and later incubated at 37°C overnight. Blood agar plates were prepared by the addition of meat extract (1%), peptone (1%), sodium chloride (0.5%), and agar (1.5%). After autoclaving, the media is cooled to RT before the addition of 5% v/v sterile defibrinated blood prior to use.

### 2.3. Screening of Microbial Lipases Production on the Agar Solid Surface

The bacterial single colonies were screened for their ability to produce lipases by using solid media containing different substrates, including Tween-20 and olive oil with phenol red. The screening assays were performed using solid media due to difficulty in the determination of lipolytic activity as the water soluble lipases catalyse reaction of only the water insoluble substrates [1, 21, 22]. The relative enzymatic activity was identified based on visual observation and measuring the formation of a clearance zone on the agar surface.

#### 2.3.1. Lipolytic Enzyme Assay Using Tween-20 Agar

Culture medium which contained peptone (1% w/v), NaCl (0.5% w/v), CaCl_2_·2H_2_O (0.01% w/v), agar (2% w/v), and Tween-20 (1% v/v) was prepared as described by Gopinath et al. [23]. Bacterial samples were then plated on the Tween-20 agar plates and incubated at 37°C overnight. The presence of lipolytic activity was indicated by a visible precipitate resulting from the calcium salt formed by the fatty acid from the hydrolysis reaction or a clearance zone around the colony due to the complete degradation of the salt of the fatty acid [23].

#### 2.3.2. Lipolytic Enzyme Assay Using Olive Oil with Phenol Red Agar

The serial diluted bacterial samples were also plated on phenol red agar and incubated at 37°C overnight. The phenol red agar plates were prepared by incorporating phenol red (0.01% w/v), olive oil (0.1% v/v), CaCl_2_ (0.1% w/v), and agar (2% w/v) [24]. Phenol red has an end point at pH 7.3-7.4, where a slight decrease in pH will turn its color from pink to yellow. The change in color of phenol red was used as an indicator for lipase activity, where lipase producing bacteria will turn the dye into yellow color.

### 2.4. Proteolytic Enzyme Assay Using Gelatin Agar

The gelatin agar plates were prepared by adding 5 mL of sterile 8% solution of gelatin (Sigma) into 100 mL of nutrient agar medium (HiMedia, India) [23]. Serial diluted bacterial samples were plated on gelatin agar and incubated at 37°C overnight. The clearance zone around the colonies indicated the presence of proteolytic activity, which was due to the complete degradation of gelatin. Aqueous saturated solution of ammonium sulfate was added on the surface of the agar for clear visualization.

## 3. Results and Discussion

**Figure 3:**
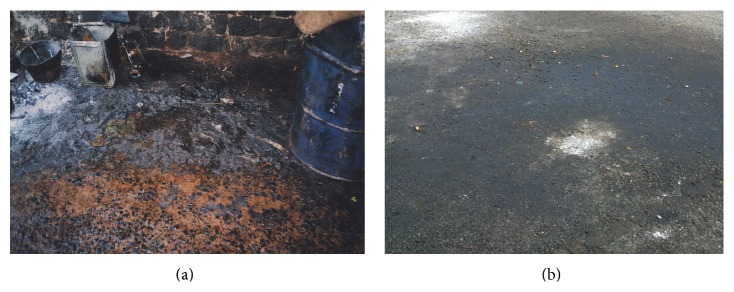
(a) Common oil spillage which may harbour certain bacteria that excrete extracellular enzymes. (b) Spot in the night market at Bandar Putra Bertam, Penang, Malaysia whereby soil sample was collected.

Isolation of extracellular enzyme-producing microorganism has garnered immense attention due to its application in numerous biotechnological processes such as detergents; textile; dairy industries; oil processing; surfactant production; and synthesis of chiral pharmaceuticals. Since there is a different requisite for the specific properties of the enzyme for each industrial application, there is a constant interest for the identification of new lipase for novel applications. Microorganisms such as bacteria, yeast, and fungi secrete certain enzymes for growth on insoluble organic substrates. For example, enzyme lipase is secreted and favored, attributable to its high reaction specificity, stereo specificity, less energy consumption, and having higher stability than the plant and animal enzymes. Previously, Gopinath et al. [23] have demonstrated the isolation of 34 fungal species from the oil-spill contaminated soils from several major cities in India and analyzed their seasonal-based changes of survival. The type of habitat for the bacteria, which harbour different substrates, can be a significant factor for the presence of the extracellular enzymes (Figure 3(a)). These enzymes also can be a potential source for economic isolation of enzymes and also a potential target for the microbial degradation-based control of oil spillage decontamination.

In this study, oil-contaminated soil samples were collected from a spot in a night market at Bandar Putra Bertam, Penang, Malaysia (Figure 3(b)). Night market was chosen as the location for sample collection as it is a very common oil-contaminated location in Malaysia and for the readiness to access the location. Samples were cultured in enrichment medium (EM1) to promote the growth of lipolytic bacteria [10] and olive oil was used as the sole carbon source (a cheaper alternative to triolein as lipase inducer).

### 3.1. Identification of Lipase Producing Bacteria

**Figure 4:**
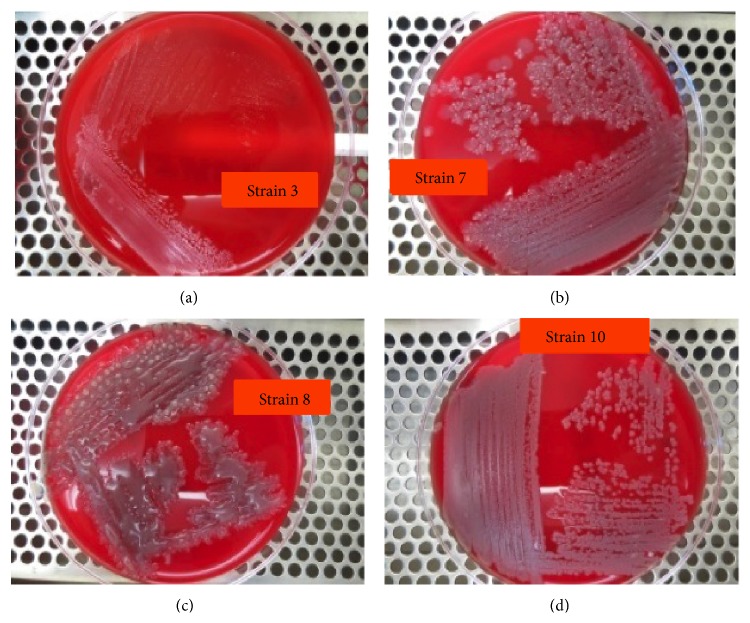
Blood agar culture of strains 3, 7, 8, and 10. Bacterial isolates grown overnight in the LB broth at 37°C are streaked onto blood agar plates (meat extract (1%), peptone (1%), sodium chloride (0.5%), and agar (1.5%)) and incubated further at 37°C overnight.

Even though 16S rDNA-based sequencing is the pragmatic approach for bacterial identification, the traditional method of Gram staining and biochemical characterization does not only aid in the identification of bacteria, but also provides information on the extracellular secretions of the bacteria. Nine of the bacteria strains (strains 2–9) were identified as Gram-positive bacilli while the other one was identified as Gram-negative bacilli (strain 1) by Gram staining studies (Table 1). Based on the differentiation via Gram stains and biochemical characterization, the Gram-positive bacteria strains were identified as* Bacillus* spp. The growth observed on the surface of the blood agar also implied the presence of Gram-positive bacteria, in this case* Bacillus* spp. (Figure 4). For the extracellular secretion analysis, the bacteria samples were subjected to lipase assays on Tween-20 with olive oil as the substrate and phenol red agar as the indicator and protease assay by using gelatin agar plates.

### 3.2. Analyses of the Lipase Activity

Tween-20, a detergent that has fatty acids (C_12_) with medium chain length, has been reported as a potential substrate for the assay of soil lipase activity [23, 25, 26]. The use of Tweens has been criticized due to the possibility of Tweens being hydrolyzed by esterases, thus resulting in false-positive results in the lipase-screening assay [27, 28]. However, Tweens are still favored as lipase substrates in screening assay due to their readiness to be incorporated into culture media and their ability to promote optimal contact between cells and/or enzymes and the substrate [28]. Seven out of the 10 bacterial strains selected showed visible precipitates on the colonies (Table 2) which could be an indication of lipolytic activity due to the release of the fatty acids from Tween-20 and their precipitation as the calcium salts [23, 26].

**Figure 5:**
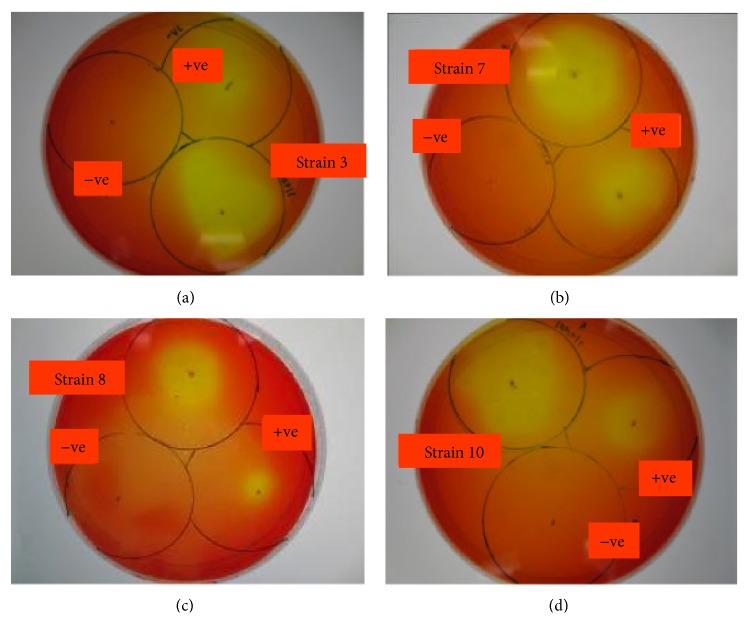
Screening of microbial lipases (strains 3, 7, 8, and 10) production on the agar plate containing olive oil as the substrate with phenol red as the pH indicator.

In order to further confirm the above determined lipolytic activity, phenol red method was carried out. Phenol red, or phenolsulfonphthalein (PSP), is a pH indicator dye that has an end point at pH 7.3-7.4 where the color is pink. When the pH gradually decreases to pH 7.0-7.1, this will result in yellow coloration. Singh et al. [24] proved that the use of phenol red in lipase assay was highly reproducible with sensitivity level as low as 0.5 p-nitrophenyl palmitate (p-NPP) enzyme units within 15 min. Therefore, by using olive oil as the lipidic substrates in phenol red agar, the presence of lipolysis activity could be indicated by the yellow coloration. This assay is based on the principle where free fatty acids were released from the bacterial lipolysis reaction [24]. All the ten bacterial strains showed lipolytic activity based on the analysis (Table 2). Four strains, 3, 7, 8, and 10, were selected for comparative lipolytic analysis by subjecting to diameter measurement of the clear zones around the colonies. Negative control (*Escherichia coli*) showed no formation of clear zone, which implied that the zone of clearance formed is accounted for by the extracellular product excreted in the sample, which is the lipase enzyme. Positive control of the strain* Pseudomonas aeruginosa* showed a clear zone of lysis, which indicated the correct formulation of the media prepared (Figure 5).

### 3.3. Proteolytic Enzyme Assay Using Gelatin Agar

**Figure 6:**
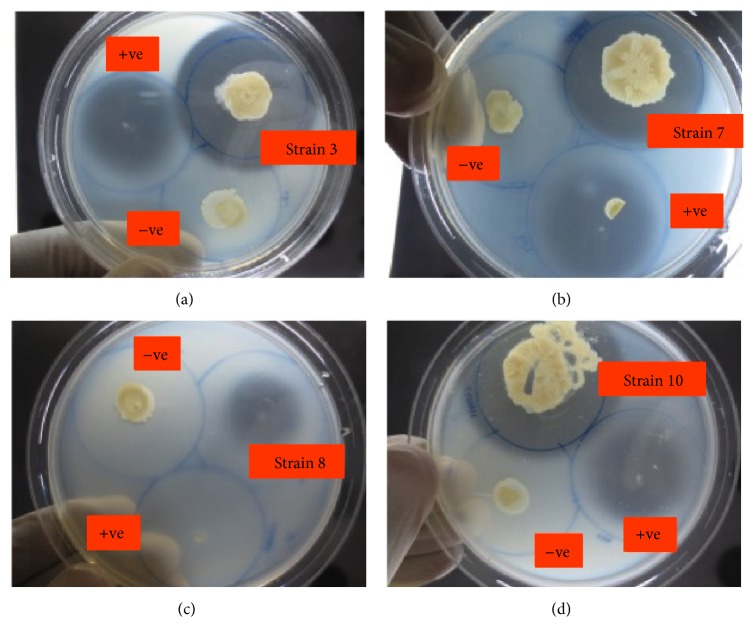
Proteolytic enzyme assay (strains 3, 7, 8, and 10) using gelatin agar. The overnight grown culture of the bacterial isolates is streaked onto gelatin agar (8% gelatin) and incubated at 37°C overnight.

Besides lipases, protease, also one of the highest value commercial enzymes, has broad applications in food and pharmaceutical and detergent industries [29]. Identification of bacterial strains with both lipases and proteases producing ability could possibly meet up the industrial demand for new sources of lipases with different catalytic characteristics [9]. The zone of clearance formed around the colonies as a result from hydrolysis of gelatin indicated that the bacteria strains are positive for proteolytic activity (Figure 6). Addition of an aqueous saturated solution of ammonium sulfate on the surface of the agar results in the opaqueness of the agar with clearer zone formation around the colonies. All the strains except for strains 1, 5, and 8 manifested proteolytic activity (Table 2). Four strains, 3, 7, 8, and 10, were selected for comparative proteolytic analysis by subjecting to diameter measurement of the clear zones around the colonies. Out of the four bacterial strains, only strains 3, 7, and 10 showed proteolytic activity, substantiated by the formation of the clear zone around the colonies. No zone of clearance was observed with negative control, which indicates that the proteolytic activity of the sample is imparted by the protease enzyme. The positive control constituted by the strain* Pseudomonas aeruginosa* showed the formation of zone of clearance, validating the authenticity of the components present in the media (Figure 6).

### 3.4. Concomitant Secretion of Lipase and Protease by* Bacillus* spp. Is a Mean of Survival

As microbes are the good source of enzyme owing to their biochemical diversity, small space for cell cultivation, and the ease of the genetic manipulation of the enzymes for the production of new enzymes for application, bacteria are widely exploited for protein production.* Bacillus subtilis, B. amyloliquefaciens*, and* B. licheniformis* are widely exploited for the purpose of protein production due to the immense fermentation nature, very high product production, and the very low level of toxic by-product.* Bacillus* strains are also able to produce a large amount of the alkaline proteases which have high significant proteolysis activity and are stable at high pH and temperature [30].

**Figure 7:**
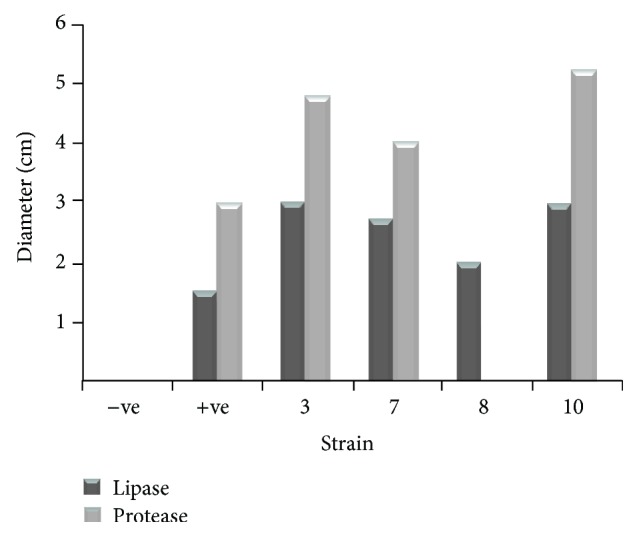
Measurement of zone of clearance of the lipolytic versus protease activity of strains 3, 7, 8, and 10. Serially diluted bacterial isolates grown overnight at 37°C were plated onto gelatin agar plate for proteolytic activity and phenol red agar for lipolytic activity.

This study has reported the secretion of lipase and protease by the* Bacillus* spp. From the measurement of the diameter of the zone of clearance, it was shown that the diameter of lipolytic activity of strain 3 has a value of 3 cm while that of protease activity has a value of 4.8 cm. On the other hand, for strain 7, the lipolytic activity exerted a zone of inhibition with the diameter value of 2.8 cm, while that of protease resulted in a value of 3.9 cm. Interestingly, strain 8 secreted no protease while the zone of clearance constituted by the lipase activity is 2 cm. Strain 10 has a value of 3 and 5.2 cm for lipolytic and protease activity, respectively. The size of the zone of inhibition is a direct indicator of the amount of enzymes excreted by the bacteria. Strain 10 has excreted the highest amount of both the lipase and protease, while strain 8 does not excrete protease (Figure 7).

The process of enzyme secretion is an energy-consuming regulatory process that is vital for the catalysis of the corresponding substrate. Moreover, the ability of the microorganism to secrete extracellular enzymes is a mean of survival, as an adaptation to the hostility of the environment. The presence of both lipid and protein in the soil has triggered the extracellular secretion of lipase and protease for the breakdown of these substrates. The accumulation of these substrates exerts pressure on the bacteria, which triggered the excretion of the enzymes. Beside substrates, other inducers for the excretion of these enzymes are the temperature, pH, sunlight, or other stress factors. These extracellular enzymes are located in the periplasmic space and are secreted depending on the sensing capacity of the microbes or quorum sensing that responds to the inducers.

### 3.5. Quorum Sensing Possibly Accounts for the Differential Secretions of the Extracellular Enzymes by the* Bacillus* spp. in the Oil-Contaminated Soil

Quorum sensing is a mechanism in bacteria whereby small molecules are excreted into the environment which aids in the adaptive response to a population [31]. The identified bacteria are Gram-positive bacteria that secrete extracellular enzymes and they are beneficial for the bacterial population [32–34]. The presence of any signalling molecules such as protein or lipid serves as the substrate or inducer for the increased secretion of both the lipase and the protease by the* Bacillus* spp. The lipid and protein molecules present in the soil initiate a mechanism that leads to the increased expression of both the lipase- and protease-encoding genes. In fact, most of the strains were reported to have secreted more protease than lipase. This could be due to the accumulated protein molecules in the soil, which are much higher than the lipid substrates. As a result, the expression of the protease-encoding genes is much more pronounced than the lipase-encoding genes. However, despite its survival, strain 8 does not secrete any protease, which is effectuated by the quorum sensing mechanism of the strain which provides less adaptive response to the presence of the substrate protein. This ignites interest to look further into the expression analysis of its protease-encoding genes, to unravel its mechanism of survival, which is unique compared to other strains.

In these findings, the excreted lipase and protease are potentially more stable than their corresponding intracellular enzymes with possible posttranslational modifications such as glycosylation and extra disulfide bond formation for increased temperature stability and higher pH resistance [35]. These enzymes also probably have enhanced stability via interaction with clay minerals, humic acid, or other compounds present in the soil [35, 36]. Hence, the oil-contaminated soil can be a promising resource of enzyme extraction, as the secreted enzymes have enhanced properties in response to the presence of the substrate in the soil. Most importantly, the isolated lipase-secreting* Bacillus* species can be a potential target strain towards the amelioration of the oil pollution.

Comprehension of the secretion and the function of the enzymes in soil are the crux of intense interest [37] which has taken an accelerating pace with the advancement of molecular and analytical techniques. As the environment is very hostile towards the stability of the enzymes, the enzymes secreted must be able to withstand the ever-present denaturation effect. Hence, this has stimulated more interest to understand the function and activity of the enzyme. A thorough insight into the function and properties of the extracellular enzymes also has many practical applications. The study shows that oil-contaminated soil is a potential location to isolate lipase producing microorganisms. With the tremendous potential of lipases in industrial applications, extensive and persistent screening for new sources of lipases with different catalytic characteristics is a matter of the utmost importance.

Environmental conditions may influence the production of the lipase and protease enzymes. Under certain conditions, the expression of the lipase- and protease-encoding genes is higher, which elevates the level of transcripts and subsequently more production of lipase/protease enzymes. Future work may focus on the identification or characterization of the regulatory elements such as transcriptional factors or small RNAs that influence the expression of the lipase-/protease-encoding genes. Identification and functional studies of these elements could render enhancement in the* in vitro* production of the recombinant lipase or protease.

## Figures and Tables

**Table 1:** Gram staining and biochemical characterization of the bacterial strains.

Strain	Gram staining	Catalase test	Coagulase test	Oxidase test	Motility test	Indole test	Citrase test	Urease test	Methyl red test	T.S.I test (slant and butt)
1	−	+	−	−	−	−	−	−	−	No changes
2	+	+	−	−	−	−	−	−	−	Acid
3	+	+	−	−	−	−	−	−	−	Acid
4	+	+	−	−	−	−	−	−	−	Acid
5	+	+	−	−	−	−	−	−	−	Acid
6	+	+	−	−	−	−	−	−	+	Acid
7	+	+	−	−	−	−	−	−	−	Acid
8	+	+	−	−	−	−	−	−	+	Acid
9	+	+	−	−	−	−	−	−	+	Acid
10	+	+	−	−	−	−	−	−	+	Acid

+, positive activity; −, no activity detected.

**Table 2:** Screening of bacteria strains for lipolytic enzyme activity.

Strain	Tween-20 agar	Phenol red agar	Gelatin agar
1	−	+	−
2	+	+	+
3	+	+	+
4	+	+	+
5	−	+	−
6	+	+	+
7	+	+	+
8	+	+	−
9	−	+	+
10	+	+	+

+, positive activity; −, no activity detected.
